# Some Observations on Typhoid Fever

**Published:** 1895-12-28

**Authors:** William Calwell

**Affiliations:** Physician to the Ulster Hospital and to the Throne Consumption Hospital; Assist. Physician to the Belfast Royal Hospital.


### SOME OBSERVATIONS ON TYPHOID FEVER.

By William Calwell, M.A., M.D., Physician to the Ulster Hospital and to the Throne Consumption Hospital; Assist. Physician to the Belfast Royal Hospital.

The following notes are based on a study of the records of over one hundred and fifty cases of typhoid fever in the Belfast Royal Hospital, and on some of my own in the Ulster Hospital for Children, and in private. They serve to illustrate some points in the natural history of the disease, and are an independent effort to arrive at the truth with regard to that most difficult of all problems—the efficacy of therapeutics.

**Occupation.**—Apprentices, shop assistants, clerks, and barmen form 40 per cent.; and of these a great number were young men away from home and new to their surroundings.

**Sex.**—Males are in considerable excess over females.

**Age.**—The statistics on this point are in very close agreement with Murchison's; the maximum is reached at 24; it is essentially a disease of adolescence. One point that struck me forcibly was the prevalence of the "gastric fever" type among children; in this type nothing ever seems to go wrong with the patient, although among the poor little care is taken; they attend regularly in the morning in the out-patient department, and I have not yet seen any of the more serious complications setting in.

**Seasonal Onset.**—The numbers are in close accordance with Murchison's. October is by far the highest month, and autumn the highest season.

**Stay in Hospital and Duration of the Disease.**—The average duration without relapse is 28·9 days, and with relapse 31·5; the extremes (omitting relapses) were 10 and 59; the average length of relapse was 18 days, with extremes of 10 and 26; the longest duration of total pyrexia was 80 days. The average stay in hospital was 45·7 days. Murchison puts the mean duration as 24·3, and the mean stay in hospital as 31·24, and his duration of relapse as 16.

**Proportion of Relapse.**—This is 14 per cent.; it was high—18·3 per cent.—in 1890, 1891, and 1892, when the mortality was low; and low in 1893 and 1894—10 per cent.—when the mortality was high.

**The Acme of the Fastigium.**—The maximum temperature was sometimes at the end of the first week, sometimes at the end of the third, and sometimes at the end of the second; but the whole was so irregular that little was to be learnt of practical use.

**Diagnosis.**—There was frequently considerable difficulty in arriving at a confident diagnosis in the first week, and, apparently, there is no method of arriving at one yet known; we may suspect, but can go no further. Ehrlich's diazo re-action was found in typhoid, but so frequently in other diseases, especially in phthisis with cavities, that it is useless.

**Complications.**—Constipation was found in 46 per cent., which is much above the statements of most books, and diarrhœa in 30 per cent.; this is, no doubt, due to the rigid restriction to milk diet. But a very interesting and important point is to be noticed; in 1890, 1891, and 1892 the constipation type was much in the ascendant (and the mortality low), whereas in 1893 and 1894 the diarrhœa type was commoner (and the mortality higher); in 1890 there were thirteen cases of constipation against two of diarrhœa, ; in 1893, fourteen cases of constipation against eighteen of diarrhœa. The constipation type, or gastric fever type, is much less dangerous as a whole than the diarrhœa type.

Hæmorrhage occurred in 8·5 per cent., with a mortality of 45 per cent.; very early "congestion" hæmorrhage is not counted. It most commonly occurred from the seventeenth to the twenty-third days, but also on the fourteenth, twenty-seventh, forty-second, and forty-third days; in four cases it took place only on one day, in five on two days, and in the rest on three days. Some diarrhœa was generally present, and it is to be noticed that diarrhœa is also commoner from the seventeenth to the twenty-second days. Constipation was present in four cases.

**Mortality.**—This was due to hæmorrhage, severe diarrhœa, general intensity of fever, with lung complications, in about equal proportions; perforation and hyperpyrexia are also noted. In 1890-92 it was 8 per cent.; in 1893-94, 18·6 per cent.; Murchison gives 15·9 per cent.

**Treatment.**—The diet is exclusively milk, about three pints, sometimes boiled, sometimes not; often diluted more or less; frequently the patient got more than three pints, even up to five without disagreement. I am strongly of the opinion that the milk should be boiled and diluted with barley water, lime water, or thin soups; as with dyspeptics, a pinch of common salt and one of baking soda will help. Weak fluid drinks may be administered with caution. For constipation, the glycerine enemata are the rule, and if carefully and not too frequently administered they cause little or no irritation, and are much pleasanter and less disturbing than the ordinary water enema, which, however, comes next in importance; I think best administered with a little baking soda, and not with soap, which hinders subsequent inspection. A very small dose of castor-oil (ʒii.), or a freshly-made rhubarb pill, has so far done no harm in the "constipation" type, but is only tried when the enema is next to impossible, or when the patient is convalescent. For diarrhœa, carefully boil and dilute the milk, and add lime (saccharated solution) and opium generally as the tincture. For hæmorrhage, prolonged constipation by large doses of opium seems the best chance. For bronchitis and pneumonia do not rush to stimulants, which are useful in tiding over a crisis, but which eventually weaken the heart; here the main point is to obtain as much nourishment as possible, and prepared foods and predigested milk (preferably with a pancreatic preparation) are to be kept in mind. I cannot satisfy myself that I have ever seen undoubted and lasting good done by ammonia, digitalis, or strychnine, although—owing to theoretical reasons—they should not be omitted, the two latter to be given hypodermically. The application of mustard night and morning to heart or chest seems to help. In the cases in which carbolic acid, corrosive sublimate, or euchloride were administered no benefit resulted; but they were too few in number to furnish sufficient data for argument. With regard to quinine, however, the same does not hold. It was given in a very large number of cases, and, in a great many, in large, continued doses, so that quantities from fifty up to three hundred grains were administered during the course, and yet no satisfactory result is forthcoming. The average length of the disease, the intensity, the complications, are all much the same as in those otherwise treated; diarrhœa, hæmorrhage, relapses, and death occur. The disease was not shortened nor modified. Cold or tepid sponging is a routine procedure in all cases; the higher the temperature, the colder the water and the more frequent the application.

